# Association between dietary acid load and risk of metabolic dysfunction-associated steatotic liver disease in patients with type 2 diabetes

**DOI:** 10.3389/fnut.2025.1494617

**Published:** 2025-05-29

**Authors:** Hesam Bakhshi, Niayesh Naghshi, Danial Fotros, Mohammadjavad Pasand, Azita Hekmatdoost, Marieh Salavatizadeh, Samira Soltanieh, Hossein Poustchi, Mohammad Ebrahim Khamseh, Zahra Yari

**Affiliations:** ^1^Department of Clinical Nutrition and Dietetics, Faculty of Nutrition Sciences and Food Technology, National Nutrition and Food Technology Research Institute, Shahid Beheshti University of Medical Sciences, Tehran, Iran; ^2^Department of Nutrition and Food Science, Wayne State University, Detroit, MI, United States; ^3^Liver and Pancreatobiliary Diseases Research Center, Digestive Diseases Research Institute, Shariati Hospital, Tehran University of Medical Sciences, Tehran, Iran; ^4^Endocrine Research Center, Institute of Endocrinology and Metabolism, Iran University of Medical Sciences, Tehran, Iran; ^5^Department of Nutrition Research, National Nutrition and Food Technology Research Institute and Faculty of Nutrition Sciences and Food Technology, Shahid Beheshti University of Medical Sciences, Tehran, Iran

**Keywords:** MASLD, diabetes, DAL, PRAL, NEAP

## Abstract

**Objective:**

Considering the high prevalence of metabolic dysfunction-associated steatotic liver disease (MASLD) among patients with type 2 diabetes and its associated complications, this study aimed to investigate the relationship between dietary acid load (DAL) and the risk of MASLD in patients with diabetes.

**Methods:**

This cross sectional study was conducted on 200 patients aged 18 to 70 with type 2 diabetes. Of whom, 133 participants were diagnosed with MASLD based on transit elastography (Fibroscan). For biochemical evaluation of liver enzymes, lipid profile, and fasting blood sugar, venous blood samples were collected after 10–12 h of fasting. Dietary acid load was determined using a 147-item food frequency questionnaire based on PRAL (potential renal acid load) and NEAP (net endogenous acid production).

**Results:**

A total of 108 women and 92 men with an average age of 52.2 years and an average body mass index of 28.8 kg/m^2^ participated in the study. After adjusting for confounders, the risk of MASLD in the third tertile of PRAL was 3.1 times higher than the first tertile (OR = 3.1, 95% CI = 1.2–7.7). After adjusting for all confounding factors, participants in the highest tertile of NEAP had nearly seven times the chance of developing MASLD compared to those in the lowest tertile, which was statistically significant (OR = 7.3, 95% CI = 2.6–20.3). Overall, the data analysis revealed a significant direct relationship between both PRAL (P trend = 0.016) and NEAP (P trend < 0.001) with the risk of MASLD.

**Conclusion:**

Our analysis revealed that a higher dietary acid load is associated with an increased risk of MASLD and liver steatosis in patients with type 2 diabetes.

## Introduction

Metabolic dysfunction-associated steatotic liver disease (MASLD) has the highest prevalence among liver diseases and affects more than a quarter of the general population (1, 2). This condition is distinguished by the accumulation of excess fat and steatosis in over 5% of liver cells, in the absence of significant alcohol consumption (3). MASLD has a broad spectrum, ranging from simple steatosis to non-alcoholic steatohepatitis (NASH), which can be associated with varying degrees of inflammation and fibrosis, eventually progressing to cirrhosis and hepatocellular carcinoma (HCC) (4). Likely symptoms of MASLD include right upper quadrant abdominal discomfort, fatigue, hepatomegaly, acanthosis nigricans, and lipomatosis, although it can be asymptomatic until advanced stages (5). Obesity, insulin resistance, diabetes, genetics, and dietary factors such as refined carbohydrates and fructose are the primary risk factors for MASLD (6, 7). The prevalence of MASLD in type 2 diabetes is estimated to be about 60%, which is almost twice that of the general population (8). Various mechanisms have been proposed to explain the association between fatty liver and diabetes. For example, insulin resistance by stimulating the enzyme lipase and releasing non-esterified fatty acids (NEFA), increases the flow of NEFA to the liver and increases the formation of triglycerides, which ultimately causes MASLD. It has been shown that adipose triglyceride lipase and hormone-sensitive lipase are the main enzymes in fat degradation. In other words, adipose tissue lipase is involved in adipose tissue lipolysis. Thus, controlling and preventing MASLD in patients with diabetes is critical (9).

The acid–base equilibrium, which is essential for the body’s metabolic health, may be influenced by the amalgamation of many dietary constituents (10). Dietary acid load (DAL) is a useful tool to assess dietary acidity (11) and is determined based on the two components of potential renal acid load (PRAL) and net endogenous acid production (NEAP) (11, 12). PRAL indicates the amount of acid and alkali produced due to dietary nutrients. If in the body, the amount of anions exceeds the amount of cations, the urinary hydrogen ion (acidic) excretion mechanism is stimulated. The total amount of excreted acid is called NEAP, which can be estimated through dietary components (13, 14) Higher PRAL and NEAP scores indicate a more acidic diet (15). In general, nutrients with acidic properties typically encompass phosphorus and protein, specially sulfur amino acids, whereas micronutrients with alkaline properties comprise calcium, potassium, and magnesium (14). A clinical trial showed that a vegan diet reduced and a meat-rich diet increased the acid load (16).

It has been reported that consumption of acidogenic foods can cause mild metabolic acidosis and inflammation in the body (17). The results of a 14-year cohort study showed a direct relationship between increased dietary acid load and the risk of type 2 diabetes (18). Similar results were published in a case–control study in 2023 (19). Also, there are studies relating DAL and MASLD. In a cross-sectional study, it was found that a high PRAL score is associated with a higher level of alanine aminotransferase (ALT) concentration and hepatic steatosis, although this relationship was seen only in women (20). Another study conducted in the Asian adult population found a direct relationship between estimated NEAP and hepatic infiltration, while no such relationship was seen with PRAL (21). A large cross-sectional study demonstrated an increasing relationship between PRAL and NEAP with MASLD and liver fibrosis (22).

The causal relationship between the acid load of the diet and the development of diseases is still not fully clear and the available results are contradictory. High occurrence of MASLD in patients with type 2 diabetes and the subsequent decline in their quality of life make it very advantageous to investigate dietary aspects that may minimize the occurrence of hepatic steatosis in these people. The current study is the first to look into a potential link between dietary acid load and type 2 diabetes patients’ risk of MASLD.

## Materials and methods

### Participants

The methodology of this study has been fully described elsewhere (23). Here, additional explanations are given in brief. This cross sectional study was conducted between April 2021 and February 2022. The study population was selected from patients aged 18 to 70 years with type 2 diabetes referring to the Institute of Diabetes and Metabolism, Iran University of Medical Sciences, Tehran, Iran. In this study, 200 patients with diabetes participated. After performing the fibroscan test, 133 patients with MASLD were identified (diagnostic criteria was the score of the controlled reduction parameter of more than 270 dB/m). In addition, at least 2 years of history of diabetes and BMI ≥ 23 were other criteria for entering the study.

Exclusion criteria included: (1) pregnancy and breastfeeding, (2) weight loss of more than 10% in the last 6 months or use of weight loss drugs or adherence to weight loss diet, (3) history of acute or chronic liver disorders (hepatitis), biliary disorders, autoimmune and hereditary diseases (hemochromatosis and Wilson’s) that affect the condition of the liver, (4) history of chronic inflammatory diseases, kidney diseases and heart failure and heart infarction, (5) history of any type of pathologically confirmed cancer or undergoing chemotherapy or radiotherapy, (6) use of insulin or anti-inflammatory drugs and toxins and drugs affecting the liver (such as nonsteroidal anti-inflammatory drugs), hormones, corticosteroids, (7) Alcohol consumption (≥21 units/week in men and ≥14 units/week in women), (8) unwillingness to cooperate or complete questionnaires and assessments. According to previous studies, the sample size was calculated to be at least 56 subjects in each group (24).

The sample size was based on the study by Mantovani et al. (24), who reported aspartate aminotransferase levels in patients with type 2 diabetes with and without MASLD as 14 ± 7 and 11 ± 3 IU/L, respectively. Considering the 95% confidence interval (CI) and 80% power of the study, and based on the following formula [(Zα + Zβ)^2^ (S_1_^2^ + S_2_^2^) / ( $\bar{X}$ _1_ - $\bar{X}$ _2_)^2^], a minimum of 56 subjects were estimated for each group.

### Ethical considerations

This study received ethical approval from the Ethics Committee of Shahid Beheshti University of Medical Sciences (Approval No: IR.SBMU.NNFTRI.REC.1400.010) on May 12, 2021. Verbal and written consent was obtained.

### Assessments

After participants joined the study, the required information about age, smoking and alcohol consumption, supplement and medication use, and the duration of diabetes was obtained through a standard general information questionnaire. For biochemical evaluation, venous blood samples were collected after 10–12 h of fasting. Fasting blood sugar (FBS), triglycerides (TG), high-density lipoprotein (HDL), serum total cholesterol (TC), serum glutamic-oxaloacetic transaminase (SGOT), and serum glutamate-pyruvate transaminase (SGPT) were measured by enzymatic methods using standard biochemical kits (Pars Azmoun Company, Iran). The between-run and within-run coefficient of variations were less than 6.2%. Low-density lipoprotein (LDL) was determined using a modified version of the Friedewald equation (25). Also, the transient elastography method (Fibroscan®) (Echosens, Paris, France) was used to evaluate the state of liver steatosis and the degree of fibrosis and based on CAP, subjects were divided into two groups, MASLD and control.

For the purpose of anthropometric assessment, a Seca 808 (Germany) digital scale with an accuracy of 100 grams was used to measure weight (kg). The weight of the participants was taken in light clothes and without shoes. Height was assessed through the utilization of a measuring tape while standing without shoes with an accuracy of 0.5 cm, and finally, weight was divided by the square of height to calculate body mass index (BMI) (kg/m^2^). Dual-energy X-ray absorptiometry (DEXA) was used to evaluate the body composition and percentage of Fat mass and Lean body mass. The International Physical Activity Questionnaire (IPAQ) was utilized to calculate the physical activity of the participants in the last 7 days (metabolic equivalent task (MET)- minutes per week).

### Dietary assessment and DAL calculation

Participants’ dietary intake was assessed using a reliable semi-quantitative food frequency questionnaire (FFQ) comprising 147 items (24), which showed information about subjects’ food intake and the average consumption of different food groups over the past year. In addition, the average intake of calories, macronutrients, potassium, calcium, phosphorus and magnesium was obtained and the ratios of total protein, animal protein, and plant protein intake to potassium intake for all participants were calculated. The interview to gather this information was carried out in person by a nutritionist who was not aware of the subjects’ condition (in terms of having MASLD). The data was then analysed using Nutritionist 4 software (First Databank Inc., Hearst Corp., San Bruno, CA, USA) that was adapted for Iranian cuisine.

Two established formulae are used to compute the PRAL and NEAP indices, which are used to assess the DAL. According to Frassetto et al.’s (12) study, to calculate NEAP, the amount of sulfuric acid production due to protein metabolism and bicarbonate production following the metabolism of intestinally absorbed potassium salts of organic acids are considered:


$$\begin{array}{l} \text{NEAP}\;(\mathrm{mEq}/\mathrm{day})=\\ \;[54.59\times \text{protein}\;(g/\mathrm{day})/\text{potassium}\;(\mathrm{mEq}/\mathrm{day})]-10.2 \end{array}$$

According to the study by Remer et al. (11) to calculate PRAL, the rate of intestinal absorption of protein, potassium, phosphate, magnesium, and calcium is considered:


$$\begin{array}{l} \text{PRAL}\;(\mathrm{mEq}/\mathrm{day})=[0.4888\times \text{protein}\;(g/\mathrm{day})]\\ +[0.0366\times \text{phosphorus}\;(\mathrm{mg}/\mathrm{day})]\\ -[0.0205\times \text{potassium}\;(\mathrm{mg}/\mathrm{day})]\\ -[0.0263\times \text{magnesium}\;(\mathrm{mg}/\mathrm{day})]\\ -[0.0125\times \text{calcium}(\mathrm{mg}/\mathrm{day})]. \end{array}$$

### Statistical analysis

All statistical analyses were performed using SPSS (SPSS Inc., version 25). The Kolmogorov–Smirnov test was performed to check the normality of the data. Participants were categorized into tertiles of PRAL and NEAP. Results are described as mean ± standard deviation for continuous variables and categorical variables were presented as numbers and percentages. The chi-square test and one-way analysis of variance (ANOVA) were used to compare categorical and continuous variables across PRAL and NEAP tertiles, respectively. A multiple logistic regression model was used to control for confounding effects of age, sex, energy intake, BMI, smoking, physical activity, duration of diabetes, FBS, TG, and TC. *p*-values less than 0.05 were considered significant.

## Results

This study included the recruitment of 200 persons diagnosed with diabetes, consisting of 108 females and 92 males. A total of 133 people were diagnosed with MASLD, while 67 participants were not. Table 1 shows the general characteristics of the participants according to the DAL tertiles, which include PRAL and NEAP. The number of patients with MASLD increased significantly with increasing levels of PRAL and NEAP (*p* = 0.030 and *p* = 0.001, respectively). There were no significant differences in age, sex, duration of diabetes, height, weight or body composition between PRAL and NEAP tertiles. Lipid profiles, FBS and liver enzymes were not different among PRAL and NEAP tertiles. CAP score, indicating the degree of hepatic steatosis, showed a significant increase with the increase of PRAL and NEAP scores (*p* = 0.029 and *p* = 0.007, respectively).

**Table 1:** Baseline general characteristics of study participants by TERTILE of DALs.

	PRAL (mEq/day)				NEAP (mEq/day)			
Variable	Tertile 1 (< −21.2) (*n* = 59)	Tertile 2 (−21.2, −6.7) (*n* = 70)	Tertile 3 (−6.7 ≤) (*n* = 71)	*p*-value	Tertile 1 (< 28.3) (*n* = 59)	Tertile 2 (28.3, 36.7) (*n* = 70)	Tertile 3 (36.7 ≤) (*n* = 71)	*p*-value
Cases of MASLD, *n* (%)	33 (25)	44 (33)	56 (42)	0.030	30 (23)	45 (34)	58 (43)	0.001
Sex (female), (%)	52	51	59	0.570	51	47	63	0.130
Age (y)	53.2 ± 8.7	51.1 ± 9.5	52.4 ± 9.6	0.448	53.5 ± 8.5	52.4 ± 9.7	51 ± 9.5	0.319
Smoker, %	20	18	15	0.753	20	20	13	0.411
Duration of diabetes (year)	8.3 ± 5.3	9.8 ± 6.4	8.5 ± 5.6	0.284	8.8 ± 5.6	9.4 ± 6.2	8.4 ± 5.6	0.619
Weight, kg	75.9 ± 12.8	79.3 ± 15.1	79.9 ± 14.9	0.266	76.8 ± 12.1	79.3 ± 15.1	79.1 ± 15.5	0.567
Height, cm	165.3 ± 10.4	165.5 ± 10	164.7 ± 9.6	0.887	165.9 ± 9.3	166.1 ± 10.6	163.7 ± 9.7	0.283
BMI, kg/m^2^	27.7 ± 3.2	28.9 ± 4.7	29.5 ± 4.5	0.057	27.9 ± 3.8	28.7 ± 4.3	29.5 ± 4.5	0.092
Fat Mass (%)	37.7 ± 7.9	38.5 ± 8.6	38.8 ± 7.4	0.785	37.3 ± 8.2	38.7 ± 8.2	38.8 ± ‌ 7.4	0.655
Lean body mass (%)	60.1 ± 9.3	58.34 ± 8.2	57.9 ± 7.1	0.427	59.3 ± ‌ 7.8	58.1 ± 7.9	58.8 ± 8.7	0.762
Physical activity (MET-min/week)	738.5 ± 1,282	856.3 ± 1,028	1,017 ± 1941	0.558	734.9 ± 857	935.1 ± 1451.9	944.9 ± 1893	0.675
FBS (mg/dl)	157.9 ± 61.5	144.9 ± 56	148 ± 61	0.442	154.6 ± 57.4	143.9 ± 52.2	151.9 ± 67.9	0.563
TC (mg/dl)	151.2 ± 39.9	145.5 ± 37.7	144.7 ± 60.9	0.658	150.1 ± 37.9	147.8 ± 40	142.4 ± 61.2	0.645
TG (mg/dl)	173.1 ± 93.8	138.1 ± 79.2	146.2 ± 86.9	0.065	164.7 ± 80.6	140.7 ± 75.8	151.6 ± 101.9	0.306
HDL (mg/dl)	50.3 ± 13.8	49.1 ± 11.9	49.1 ± 12.9	0.838	52.6 ± 15.6	49.1 ± 10.4	47.1 ± 12.1	0.054
LDL (mg/dl)	78.3 ± 26.9	76 ± 27.5	67.7 ± 25.2	0.056	76.8 ± 26.8	78.7 ± 28.2	66.1 ± 24.2	0.012
SGOT (U/L)	20.6 ± 9.6	20.3 ± 8.4	20.6 ± 8.9	0.970	20.4 ± 9.8	20.7 ± 7.7	20.4 ± 9.4	0.971
SGPT (U/L)	19.5 ± 10.6	19.1 ± 10.8	18.1 ± 7.9	0.688	20.5 ± 12.3	18.1 ± 9	18.2 ± 7.9	0.317
CAP score (db/m)	286.8 ± 45.6	293 ± 47.9	307.1 ± 41.5	0.029	285.6 ± 44.2	291.8 ± 49.1	309.4 ± 40	0.007

The results are described as mean ± standard deviation (ANOVA test) or number (%) (Chi-square test). PRAL, potential renal acid load; NEAP, net endogenous acid production; BMI, body mass index.

Table 2 displays the dietary intakes of the participants across the PRAL and NEAP tertiles. Individuals with higher potassium intake had significantly lower PRAL and NEAP (*p* = 0.004 and *p* = 0.001, respectively). Additionally, participants in the third tertile of PRAL and NEAP had higher protein and phosphorus consumption compared to those in the first tertile, though this difference was not significant. In terms of food groups, participants in the third tertile of PRAL and NEAP consumed significantly less vegetables than those in the first tertile. Similarly, fruit consumption had an inverse relationship with increasing PRAL and NEAP, although this relationship was significant only for NEAP (*p* < 0.001). Participants in the third tertile of PRAL and NEAP consumed more grains than those in the first tertile, with the difference only significant for PRAL (*p* = 0.007). There was no significant relationship between red meat or dairy products and DAL tertiles. Overall, there was a significant direct relationship between the ratio of plant and animal protein intake to potassium intake, with the increase in PRAL and NEAP (*p* < 0.001).

**Table 2:** Dietary intakes of patients across tertiles of dietary acid–base load.

	PRAL (mEq/day)				NEAP (mEq/day)			
Variable	Tertile 1 (< −21.2) (*n* = 59)	Tertile 2 (−21.2, −6.7) (*n* = 70)	Tertile 3 (−6.7 ≤) (*n* = 71)	*p*-value	Tertile 1 (< 28.3) (*n* = 59)	Tertile 2 (28.3, 36.7) (*n* = 70)	Tertile 3 (36.7 ≤) (*n* = 71)	*p*-value
Calorie (Kcal/d)	2,623 ± 747	2,394 ± 731	2,375 ± 721	0.968	2,442 ± 739	2,454 ± 750	2,475 ± 733	0.117
Carbohydrate (g/d)	364 ± 96	337 ± 115	319 ± 104	0.961	336 ± 100	341 ± 118	338 ± 101	0.066
Protein (g/d)	85 ± 31	84 ± 31	94 ± 37	0.111	82 ± 32	86 ± 31	94 ± 35	0.147
Fat (g/d)	87 ± 31	76 ± 28	76 ± 24	0.861	79 ± 28	81 ± 29	78 ± 26	0.033
Phosphorous (mg/d)	1,579 ± 545	1,625 ± 631	1,686 ± 605	0.382	1,541 ± 562	1,676 ± 645	1,666 ± 571	0.595
Potassium (mg/d)	5,232 ± 1,573	4,143 ± 1,344	3,829 ± 1,538	0.004	4,903 ± 1801	4,305 ± 1,546	3,981 ± 1,341	0.001
Calcium (mg/d)	1,136 ± 452	1,104 ± 438	1,087 ± 413	0.708	1,078 ± 448	1,143 ± 457	1,098 ± 398	0.824
Magnesium (mg/d)	518 ± 180	465 ± 175	486 ± 216	0.878	499 ± 208	482 ± 183	486 ± 190	0.304
Food groups								
Grains (g/d)	413 ± 165	456 ± 182	483 ± 202	0.007	393 ± 176	459 ± 178	496 ± 192	0.099
Fruits (g/d)	562 ± 218	451 ± 203	367 ± 193	0.083	490 ± 225	462 ± 220	403 ± 200	<0.001
Vegetables (g/d)	464 ± 198	381 ± 192	334 ± 193	0.003	454 ± 215	384 ± 184	333 ± 189	0.001
Red meat (g/d)	24 ± 17	25 ± 24	26 ± 23	0.052	20 ± 12	26 ± 22	29 ± 26	0.794
Dairy products (g/d)	363 ± 236	353 ± 231	323 ± 196	0.354	348 ± 232	372 ± 229	318 ± 201	0.552
Legumes and nuts (g/d)	50 ± 36	38 ± 24	35 ± 27	0.494	38 ± 31	44 ± 28	40 ± 30	0.012
protein to potassium ratio	0.62 ± 0.09	0.78 ± 0.08	0.94 ± 0.16	<0.001	0.64 ± 0.12	0.78 ± 0.05	0.94 ± 0.18	<0.001
Animal protein to potassium ratio	0.26 ± 0.08	0.35 ± 0.1	0.46 ± 0.21	<0.001	0.27 ± 0.09	0.35 ± 0.1	0.45 ± 0.22	<0.001
Plant protein to potassium ratio	0.36 ± 0.1	0.43 ± 0.11	0.51 ± 0.19	<0.001	0.36 ± 0.12	0.43 ± 0.11	0.51 ± 0.17	<0.001

PRAL, potential renal acid load; NEAP, net endogenous acid production. The results are described as mean ± standard deviation using ANOVA test.

Table 3 illustrates the correlation between DAL and the risk of MASLD. According to model 1, in which only age and gender were adjusted for, participants in the second (OR = 1.58, 95% CI = 0.76–3.3) and third (OR = 2.63, 95% CI = 1.24–5.56) tertiles of PRAL and NEAP had a higher risk of MASLD than the first tertile (*p* = 0.012). Following adjustments for age, sex, energy intake, BMI, smoking, physical activity, duration of diabetes, FBS, TG, and TC, model 3 showed a statistically significant 3.1-fold increase in the probability of MASLD in the third tertile of PRAL compared to the first tertile (OR = 3.1, 95% CI = 1.2–7.7). In the second tertile of PRAL, this risk was twice that of the first tertile, although it was not statistically significant (OR = 2, 95% CI = 0.8–4.99). Participants in the highest tertile of NEAP had a nearly sevenfold increased risk of developing MASLD compared to those in the lowest tertile, which was statistically significant (OR = 7.3, 95% CI = 2.6–20.3). Overall, a significant direct relationship between PRAL and NEAP with the risk of MASLD was observed in the data analysis (P trend = 0.016 and P trend <0.001, respectively).

**Table 3:** Odds and 95% confidence interval for occurrence of the MASLD in each tertile categories of DAL.

	Tertiles of dietary acid load			*P* trend
PRAL	T1 (< −21.2)	T2 (−21.2, −6.7)	T3 (−6.7 ≤)	
No. of cases	33	44	56	0.030
Model 1	ref	1.58 (0.76, 3.3)	2.63 (1.24, 5.56)	0.012
Model 2	ref	1.49 (0.66, 3.37)	2.17 (1.03, 5)	0.049
Model 3	ref	2 (0.8, 4.99)	3.1 (1.2, 7.7)	0.016

	Tertiles of dietary acid load			*P* trend
NEAP	T1 (< 28.3)	T2 (28.3–36.7)	T3 (36.7 ≤)	
No. of cases	30	45	58	0.001
Model 1	ref	1.7 (0.88, 3.66)	4.2 (1.8, 9.3)	<0.001
Model 2	ref	1.8 (0.79, 4)	4.3 (1.74, 10.8)	0.002
Model 3	ref	2.2 (0.9, 5.3)	7.3 (2.6, 20.3)	<0.001

Based on multiple logistic regression model. Model 1: adjusted for age and sex; Model 2: additionally adjusted for energy intake, BMI, smoking, physical activity; Model 3: additionally adjusted for duration of diabetes, FBS, TG, TC.

## Discussion

This cross-sectional research was conducted to examine the correlation between dietary acid load and MASLD in individuals with type 2 diabetes. The study concluded that with an increase in DAL, as evaluated by PRAL and NEAP, the risk of developing MASLD and hepatic steatosis increases significantly in patients with diabetese. This significant incremental relationship became stronger after adjusting for all confounding factors. These findings confirm the hypothesis of the study.

Several studies aimed at investigating the association between DAL and the occurrence of MASLD have been carried out, yielding diverse results. A cross-sectional study conducted by Cheng et al. (22) using NHANES data on a large population revealed a direct and non-linear relationship between DAL and MASLD. Another cross-sectional study carried out among the Chinese adult population in 2015 found a significant direct relationship between the likelihood of MASLD and NEAP but no significant relationship with PRAL. One limitation of this study was that it assessed participants’ food intake only over the past week, whereas our study examined the average food intake over the past year (21). Alferink et al. (26) reported that the highest odds of MASLD was associated with an acidic diet, while the lowest probability was linked to a balanced diet, although this association was not linear. This finding generally indicates that a diet based on plant protein and rich in fruits, vegetables and potassium has better results than a diet rich in animal protein for the prevention of MASLD (26). Sulfur-containing amino acids, such as methionine and cysteine, mainly found in animal proteins, undergo oxidation to form sulfate and ultimately hydrogen ions, therefore forming an acidic molecule. In contrast, plant proteins mainly contain glutamate, which does not produce hydrogen ions during metabolism. Fruits and vegetables are known as acid-neutralizing compounds due to their abundance of potassium, citrate, and malate (27, 28).

A study by Krupp et al. (20) found a significant relationship between increased PRAL and elevated ALT enzyme levels and hepatic steatosis index (HSI) in women, but this relationship was not observed in men. A limitation of this study was the absence of a reliable method for evaluating liver steatosis, such as FibroScan (20). In a case–control study, Emamat et al. (29) evaluated the relationship between PRAL and the risk of MASLD. The findings showed no significant difference between the highest and lowest quintiles of the PRAL index. However, a U-shaped correlation between PRAL and the likelihood of MASLD was observed, with the middle quintiles having a lower risk of developing MASLD. As a result, a diet balanced in terms of acid–base was associated with a lower risk of developing MASLD (29). A cross-sectional study found that people with higher PRAL and NEAP had a slightly higher chance of MASLD, but the result was not statistically significant. An inherent constraint of this research was the categorization of PRAL and NEAP into just two distinct groups: high and low (30). The majority of the published research yielded comparable findings and aligned with our own analysis, indicating that an elevated DAL may serve as a risk factor for MASLD.

The mechanism of association between DAL and MASLD is not yet clearly defined. An increase in DAL can lead to the disruption of the body’s acid–base balance and the induction of mild metabolic acidosis (MA), which results from the increase in non-carbonic acids due to the consumption of animal proteins and the decrease in citrate and bicarbonate due to inadequate intake of fruits and vegetables (12). Chronic MA caused by acidogenic foods may lead to growth hormone (GH) resistance in the body, which can affect systemic metabolism and immune status and modulate the activation of hepatic stellate cells and eventually result in liver cell damage and MASLD (31, 32). Overall, there is a substantial relationship between type 2 diabetes and MASLD, which is usually due to insulin resistance and obesity (33). Various mechanisms, including increased NEFA levels, oxidative stress, mitochondrial dysfunction, and the release of inflammatory mediators are involved (34). Consequently, factors that exacerbate insulin resistance and obesity also elevate the risk of MASLD. A case–control study by Ozturk et al. (19) in 2023 included 204 participants, 92 of whom had type 2 diabetes while the remaining did not. The study revealed that individuals in the top tertile of PRAL and NEAP were more likely to develop type 2 diabetes and insulin resistance compared to those in the lower tertile. Therefore, a diet with a high acid load is associated with an increased risk of insulin resistance (19). A similar result was seen in another study (35). Jayedi et al. (36) confirmed a direct relationship between elevated DAL and increased risk of type 2 diabetes through a meta-analysis.

As mentioned, an increase in PRAL and NEAP can lead to mild MA. It has been observed that MA can reduce the phosphorylation of protein kinase B, alter the expression of the insulin receptor, decrease urinary citrate secretion, increase magnesium excretion, and eventually cause insulin resistance (37, 38). Another possible mechanism in the relationship between diabetes and DAL is insulin resistance which can be considered a physiological response. By decreasing insulin sensitivity, muscle protein becomes available for breakdown and the formation of ammonium as a neutralizing buffer (39). It has also been observed that in MA, glucocorticoids and cortisol levels increase as insulin antagonists, while adiponectin levels decrease as insulin sensitizers (40, 41).

There are several studies regarding DAL and obesity. In 2018, Abbasalizad Farhangi et al. (42) performed a meta-analysis and concluded that an increase in PRAL and NEAP increases the risk of obesity and the concentration of TG. The relationship between PRAL and metabolic syndrome (MetS) in obese and overweight women was examined in another investigation. It was found that people with high PRAL had a higher chance of developing MetS, but this finding was not statistically significant (43). Additionally, Rajaie et al. (44) showed in a cross-sectional study that there is no significant relationship between DAL and MetS. The risk of hypertriglyceridemia, however, increased in women when DAL levels rose (44). The process by which a rise in DAL results in elevated TG levels is still not completely understood, but it may involve the development of insulin resistance, raised cortisol levels and its lipolytic activity, which can ultimately result in higher TG levels and accumulation in the liver (45).

The strengths of our research lie in the evaluation of food consumption via the use of a validated FFQ and in-person interviews carried out by an expert nutritionist. Additionally, MASLD was diagnosed using a fibroscan. However, our study also had limitations. First, as a cross sectional study, it is not possible to generalize cause-and-effect relationships from our results. So since the cross-sectional design of our study does not allow for causal inferences, and further longitudinal or experimental research is needed to confirm these findings and elucidate the mechanisms involved. Moreover, recall bias and measurement error are unavoidable errors in assessment of dietary intake.

In conclusion, this study showed that an increase in DAL is associated with a higher risk of MASLD and liver steatosis in patients with type 2 diabetes. Because of the high prevalence of MASLD in type 2 diabetes, prospective studies with long-term follow-up are needed to better understand the role of dietary acid load in the development of MASLD among other populations.

## Data Availability

The original contributions presented in the study are included in the article/supplementary material, further inquiries can be directed to the corresponding author.
